# Metabolism Predicts Ecological Response to Warming

**DOI:** 10.1371/journal.pbio.1000180

**Published:** 2009-08-25

**Authors:** Liza Gross

**Affiliations:** Senior Science Writer/Editor, PLoS Biology, Public Library of Science, San Francisco, California, United States of America

**Figure pbio-1000180-g001:**
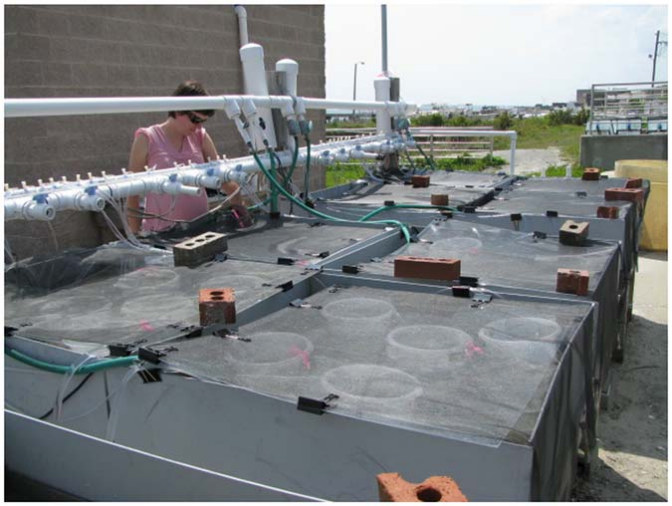
To test the effects of temperature on food web structure and productivity, Mary O'Connor (above, checking temperatures) and colleagues placed five microcosms of food webs (shielded from full sunlight and UV) in eight independent water tables, each filled with a temperature-conditioned water bath.

[Fig pbio-1000180-g001]While politicians like United States Representative Michele Bachmann (R-Minn.) rail against efforts to curb human contributions to global warming—she thinks carbon dioxide, a “natural by-product of nature,” could not possibly be harmful—scientists are documenting the damage. Numerous studies describe how climate change is threatening the persistence of a broad range of plant and animal species across diverse taxa, geographic regions, and trophic levels, from the polar bear at the top of the food chain to the shrimp-like krill at the bottom.

As they catalog the ecological casualties of a rapidly warming world, researchers are also searching for general effects of climate change to help them predict and mitigate its consequences. The search has not been easy. Many of the documented impacts reflect species' life history—upset synchrony between the peak food needs of newly hatched birds and the peak availability of their traditional insect diet, for example—marked by idiosyncrasies that defy generalization. What's more, field studies have yielded conflicting results, with warming causing significant effects on food webs in some regions but not in others. Complicating matters, experiments that test how temperature affects food web dynamics—an approach that would help validate general predictions—are rarely done.

Undaunted by such challenges, Mary O'Connor and colleagues tested the effects of temperature on an experimental marine food web in a new study in this issue of *PLoS Biology*. The authors provide empirical evidence that suggests general ecological consequences of climate change do exist. They show that phytoplankton, the primary producers at the bottom of the marine food web, and their zooplankton predators respond differently to increased temperature. These individual metabolic responses impact food webs in a predictable way that seems to depend on resource availability.

Understanding how climate change will affect ocean communities is especially important, not only because the seas account for 16% of the protein humans consume but also because they play a major role in the global climate system. Interactions between the vast communities of microbes and tiny animals that inhabit the lowest reaches of the food web may determine whether the oceans will sequester or boost carbon dioxide emissions.

In conventional food web theory, consumer productivity (in oceans, zooplankton and fish biomass) is controlled by primary productivity (phytoplankton biomass), which depends on nutrient availability. In this model, increased productivity and relative abundance of autotrophs, which take up carbon using light, would boost carbon dioxide uptake throughout the food web, offsetting rising carbon emissions. But temperature's impact on food webs is complex. Temperature affects metabolism as well as nutrient availability, but both temperature and nutrient sources fluctuate with regional currents, air temperatures, and the seasonal upwelling of the deep, cold, nutrient-rich water that recycles resources to photosynthetic autotrophs at the sea surface.

In an alternative framework, understanding how climate change affects food web productivity (the rate of production of all biomass) and structure (relative abundance of trophic levels) all boils down to metabolism. In the metabolic theory of ecology, the flow of energy and materials in an ecosystem can be determined by individual organisms' metabolic rates, which vary with temperature and body size. Though nutrient availability constrains primary production, temperature affects both primary (autotrophic) and consumer (heterotrophic) production by influencing their respective metabolic processes, photosynthesis and respiration. The theory predicts that consumers would have more control over food web dynamics under global warming because respiration is more sensitive than photosynthesis to changing temperature. Thus, O'Connor and colleagues reasoned, temperature-induced metabolic effects should differ from and complement nutrient constraints.

To investigate the effects of temperature and nutrient availability on food web structure and productivity, O'Connor and colleagues collected autotrophic phytoplankton and heterotrophic zooplankton and bacteria from their local estuary in North Carolina. The authors used the samples to create outdoor microcosms of food webs and exposed them to four temperature levels and two nutrient scenarios, mirroring the estuary's seasonal conditions.

As metabolic theory predicts, the authors found that small temperature increases shifted the balance of biomass toward consumers, resulting from different temperature-induced responses in resource use, growth, and reproduction between producers and consumers. Simply put, grazing outpaced primary production. They also observed an overall decrease in biomass, again supporting greater consumer control of food web structure as a consequence of warming. If warming had disproportionately increased primary productivity, the authors explain, total biomass would have increased.

Both food web structure and productivity were limited by nutrient availability. Adding nutrients favored consumer control and increased total biomass, which decreased with warming. Restricting nutrients, on the other hand, led to reduced biomass, and consumers lost their preponderant position. These findings may explain why field studies have reported marked effects of warming in some settings but not in others. Food webs in nutrient-poor regions may be more resilient to warming because nutrient availability will constrain primary production and maintain normal trophic structure. But in nutrient-rich regions, the authors caution, warming could disrupt this balance and have “dramatic effects on trophic structure, primary productivity, and standing biomass.”

As with any experiments that simplify nature to understand it, how well these results describe ecological conditions remains to be seen. They do, however, provide experimental evidence that universal constraints on individual metabolism can predict general responses to warming. And with the International Panel on Climate change estimating as much as a 7.2 degree Fahrenheit increase in global temperatures by the end of the century, researchers need every tool at their disposal to spot early signs of species and communities in distress.

**O'Connor MI, Piehler MF, Leech DM, Anton A, Bruno JF (2009) Warming and Resource Availability Shift Food Web Structure and Metabolism. doi:10.1371/journal.pbio.1000178**

